# The Dynamics of the Epileptic Brain Reveal Long-Memory Processes

**DOI:** 10.3389/fneur.2014.00217

**Published:** 2014-10-24

**Authors:** Mark J. Cook, Andrea Varsavsky, David Himes, Kent Leyde, Samuel Frank Berkovic, Terence O’Brien, Iven Mareels

**Affiliations:** ^1^Department of Medicine, St. Vincent’s Hospital, University of Melbourne, Fitzroy, VIC, Australia; ^2^Department of Electrical and Electronic Engineering, University of Melbourne, Fitzroy, VIC, Australia; ^3^Neurovista Corporation, Seattle, WA, USA; ^4^Department of Medicine, Austin and Repatriation Medical Centre, University of Melbourne, Fitzroy, VIC, Australia; ^5^Department of Medicine, Royal Melbourne Hospital, University of Melbourne, Fitzroy, VIC, Australia

**Keywords:** epilepsy, long-range memory, power-law phenomena, neural dynamics in cortical networks, seizure clustering

## Abstract

The pattern of epileptic seizures is often considered unpredictable and the interval between events without correlation. A number of studies have examined the possibility that seizure activity respects a power-law relationship, both in terms of event magnitude and inter-event intervals. Such relationships are found in a variety of natural and man-made systems, such as earthquakes or Internet traffic, and describe the relationship between the magnitude of an event and the number of events. We postulated that human inter-seizure intervals would follow a power-law relationship, and furthermore that evidence for the existence of a long-memory process could be established in this relationship. We performed a *post hoc* analysis, studying eight patients who had long-term (up to 2 years) ambulatory intracranial EEG data recorded as part of the assessment of a novel seizure prediction device. We demonstrated that a power-law relationship could be established in these patients (β = − 1.5). In five out of the six subjects whose data were sufficiently stationary for analysis, we found evidence of long memory between epileptic events. This memory spans time scales from 30 min to 40 days. The estimated Hurst exponents range from 0.51 to 0.77 ± 0.01. This finding may provide evidence of phase-transitions underlying the dynamics of epilepsy.

## Introduction

Epilepsy is a common and serious neurological disorder characterized by recurrent seizures. Though cycles of seizure activity associated with biological rhythms (circadian and menstrual) have long been recognized, Poisson processes have been felt to describe the pattern of seizure occurrence, with departures perhaps explained by external factors (1). Many authors have noted more variability in seizure frequency than would be expected if their distribution followed a simple Poisson model, with overdispersion in series of seizure counts (2–6).

Identification of a *power law* in epileptic inter-event times has received considerable interest (7). Power laws describe a relationship between quantities, where the frequency of an event varies as a power of another feature of the event, such as size. In such a relationship, small events occur very more frequently than large events, although the probability of the large events is non-trivial. The existence of power-law relationships is necessary for certain types of system behavior, such as critical dynamics, to exist. By itself, the presence of a power law is not a sufficient condition to prove critical system behavior, but its absence is strong evidence against such dynamics.

A *long-memory process* (or *long-range-dependent* process) is another type of dynamic behavior that can be identified by the existence of a power law in the higher order statistics of a system. This describes a situation where the decay of dependence of system dynamics on past events is slower than exponential, and correlation of events extends far beyond immediate values. This contrasts with short memory processes in which the system dynamics can be described using a short and finite memory. A parameter typically used to characterize the length of this dependence is the *Hurst exponent* (*H*). A value of *H* = 0.5 defines a system that is random (i.e., no dependence), and 0.5 < *H* < 1 defines a positive correlation, or clustering of extremes, so that long intervals are likely to be followed by long intervals, and short intervals by short intervals. Values of 0 < *H* < 0.5 imply anti-correlation, and *H* > 1 implies non-stationarity in the data. We have previously demonstrated the existence of long-memory processes in epileptic seizures in animal and limited human datasets (8).

Recognizing and understanding any long-term dynamic processes underlying epilepsy would have significant implications for its management; however, the data required for the estimation of this have not previously been available. Human scalp EEG data are easily obtainable, but has poor localization properties that obscure seizure initiation dynamics (8–10), and are typically only very short term (~1 day duration). Intracranial EEG (iEEG) improves localization, but such limited duration (~1 week) records are obtained during pre-resection assessment of hospitalized patients, under artificial circumstances of sleep and medication withdrawal, and show different seizure dynamics to those observed in ambulatory recordings.

We present here a study of the dynamics of epileptic seizure generation using intracranial, ambulatory, human EEG data with continuous records up to 2 years duration. This unique dataset was acquired for the purpose of epileptic seizure anticipation (11). To our knowledge, this is the first time data of this nature has been available for the study of epilepsy. The volume and uniqueness of the data make the possibilities for analysis immense, but we restrict our work here to the study of the distribution and correlation of seizure event times.

### Subjects

Data acquired for a clinical feasibility study of a seizure prediction device involving long-term implantation of iEEG recording electrodes were used. More details of the study can be found in Cook et al. (11). Subjects were selected primarily on the basis of medically refractory nature, with 2–12 reported seizures/month, as confirmed through patient diaries. Approval for the study was obtained though the Human Research Ethics Committees of the participating clinical centers. All subjects gave written informed consent to participate prior to any study procedures being performed.

Seventeen subjects were enrolled from the three tertiary referral epilepsy centers comprising the Melbourne University Epilepsy Group. Two of these elected to pursue other treatment options prior to implantation, and so are not included in any further results. These adult subjects were selected chiefly on the basis of suitable seizure frequency (2–12 seizures/month) and all had a level of independence sufficient to make the device useful in the management of daily activities. Nine males and six females with a mean age of 44.5 years (range 20–62 years) were implanted. Six subjects had undergone previous epilepsy resective surgery and one had used vagus nerve stimulation (VNS), which was explanted at the time of predictive system implant.

Subjects meeting inclusion/exclusion criteria were implanted with the SAS and initially entered a Data Collection Phase, where the hand-held device remained passive (where no advisories were given to the subject) and iEEG data was collected. When sufficient data was obtained, a subject-specific algorithm was created. The algorithm was then evaluated against minimum performance criteria and if satisfactory, the subject entered the Advisory Phase. In the Advisory Phase, the algorithm was enabled to provide visual and audible advisories to the subject. Throughout the study, ambulatory iEEG data were analyzed for seizure statistics and other relevant electroencephalographic events. Subjects served as their own controls for the purpose of evaluating study outcomes.

### Procedures

The major components of the implanted seizure prediction system are: (1) the implantable lead assemblies, (2) the implantable telemetry unit, (3) the external hand-held personal advisory device, and (4) the external charging accessory. In addition to these components, a cluster-computing system and associated software was used to configure algorithms for each individual subject.

**Figure 1:**
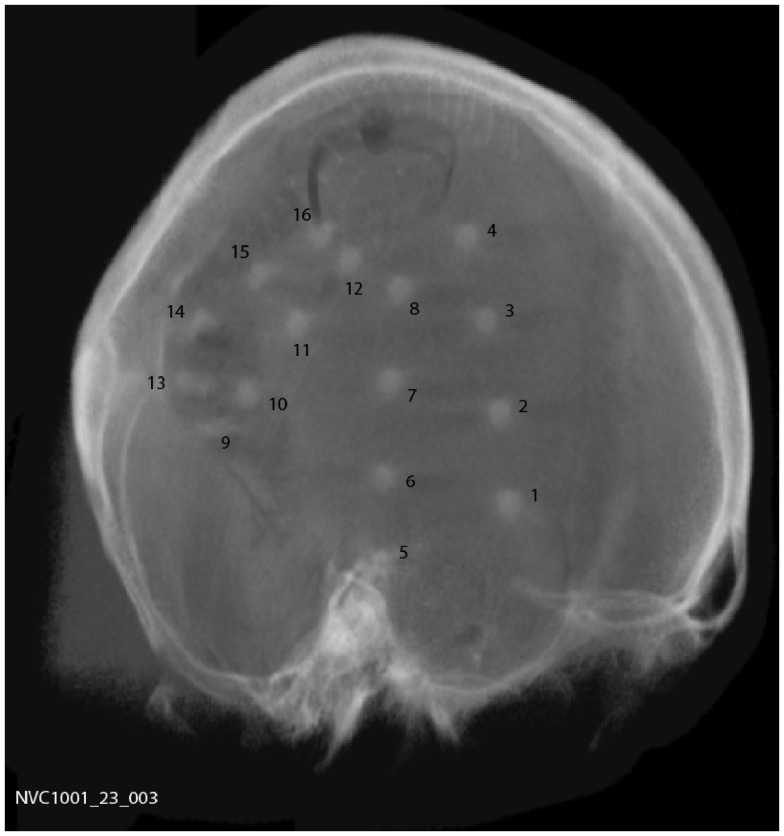
**Plain skull radiograph of subject post implantation, showing typical implantation scheme**.

A total of 15 subjects were implanted with the device, 9 males and 6 females with a mean age of 44.5 years (range 20–62). Two silicone implantable lead assemblies, each with eight platinum iridium contacts distributed across two electrode arrays (16 electrodes in total), were used to collect iEEG on the cortical surface. Leads were placed regionally, unilaterally over the quadrant believed to contain the epileptogenic zone, as determined by prior EEG studies, imaging studies, and/or seizure etiology, via a small craniotomy or through prior craniotomy sites if surgery had been performed in the past. A typical implantation scheme is shown in Figure 1. For those subjects diagnosed with bilateral temporal lobe onset seizures, leads were placed over the hemisphere that generated the most frequent, stereotypical seizures. System operation and integrity was verified prior to wound closure. The leads were tunneled down the neck and terminated at a subclavicularly placed, titanium encased, hermetically sealed, implantable telemetry unit, which sampled 16 channels of iEEG acquired at 400 Hz and wirelessly transmitted it to an external, hand-held personal advisory device. The external, hand-held personal advisory device received the telemetered iEEG and stored iEEG on standard flash memory cards for subsequent analysis. An important component was that it also supported audio recordings, both manually triggered by the subject for diary purposes, and also automatically activated when a seizure was detected by the system to aid in establishing a clinical correlate with iEEG activity. The duration of implantation varied between ~0.5 and ~1.8 years.

The clinical study algorithm utilized a layered structure consisting of a filtering layer, a feature extraction layer, and a classification layer. Each layer could be configured using a number of subject-specific parameters that were created as part of an algorithm training process.

The filtering and feature extraction layers were used to implement a form of spectral analysis. Input signals were filtered by a collection of octave-wide digital filters covering the range from 2 to 128 Hz. A wide band filter complemented these filters and optional notch filters designed to eliminate interference from AC-mains sources. Filter outputs could be analyzed for average energy or line-length over a 5 s analysis window. These outputs could be normalized using previously derived parameters or by other signals. The combination of 16 available iEEG input channels and many different filtering options created a list of several 100-candidate features. During algorithm training, the list of candidates was analyzed to find the best 16 features. This 16-dimension feature vector was then passed to the classification layer.

The classifier design was intended to create a computationally efficient implementation that was functionally similar to a k-nearest neighbor (kNN) classifier. This was accomplished through use of a partitioning approach that reduced the need to search through long lists of training data points. The classifier output was filtered, thresholded, and then passed to the user interface state machine used to control the three likelihood indicators. The main function of the state machine was to latch a condition in the case where the classifier output was repeatedly alternating across a decision threshold, preventing rapidly changing advisories and possible subject confusion.

To support the study, a revised cluster-computing environment was developed. The main function of this new cluster computer was to train subject-specific algorithms to generate the required algorithm configuration parameters. The system could also apply hold-out or cross validation quasi-prospective methods to provide an estimate of algorithm performance needed to inform the decision to proceed into the Advisory Phase of the study. The system could also be used to calculate algorithm performance using prospective data collected during the Advisory Phase.

Post processing was applied to the iEEG data to determine the time and the length of both clinical and sub-clinical seizures (defined as electroencephalographic events with no clinically relevant manifestation). Study investigators annotated the iEEG acquired and events were detected by a validated seizure detection algorithm based on an unsupervised learning approach that identifies statistically significant outliers in iEEG features associated with seizures (12). Accumulated iEEG was annotated by clinical staff and verified by study investigators utilizing subject diaries, hand-held audio recordings, and a seizure detection algorithm (12). Events were categorized as clinically reported, and found in EEG (type 1), events not clinically reported, but found in EEG, and having a similar envelope to type 1 events (type 2), and those not clinically reported, but found in EEG, without evidence of clinical manifestations (type 3). Events clinically reported but without EEG changes were excluded. A large number of events were necessary for accurate analysis, and so the 8 subjects who had more than 400 recorded events were studied. The inter-event intervals, defined as the time between the onset of one seizure and the onset of the next were used for analysis. To estimate the Hurst exponent, point processes were generated by quantizing the timescale to 1-min resolution, and the presence or absence of seizure onset at a particular time was represented with the value of 1 or 0, respectively. A summary of all data included for analysis can be found in Table 1.

**Table 1:** **Summary of data**.

	P1	P2	P3	P4	P5	P6	P7	P8
Age	22	52	48	51	50	53	43	50
Sex	F	M	M	F	F	F	M	M
Epileptogenic zone	PT	FT	FT	OP	FT	FT	T	T
AED’s	CBZ, LTG, PHT	CBZ, CLZ, LEV	CBZ, LEV	CBZ	LEV, OXC, ZNS	LCM, PHT, PRP	LTG, LCM, PHT, RTG	CBZ, CLZ, LEV, LCM
Record length (days)	523	182	504	305	313	646	650	618
Total seizures	1569	574	446	750	1088	479	4561	985
Median ISI	3 min	41 min	11.5 h	3 h	21 min	13 h	5 min	1 h

*AED’s: CBZ, carbamazepine; CLZ, clonazepam; LCM, lacosamide; LEV, levetiracetam; LTG, lamotrigine; OXC, oxcarbazepine; PHT, phenytoin; PRP, perampanel; RTG, retigabine; ZNS, zonisamide; epileptogenic zones: FT, frontotemporal; OP, occipitoparietal; PT, parietal-temporal; T, temporal*.

## Materials and Methods

### Estimating power laws

Mathematically, a quantity *x* follows a power law if it is drawn from a probability distribution *f*(*x*) ∝ *x*^β^. The scaling exponent β is a constant that can be estimated as the (linear) gradient of the log–log plot log[*f*(*x*)] = β log(*x*) + c. This estimate is highly susceptible to errors when the dataset is not ideal, such as brevity, non-stationarity, or inaccurate records.

### Estimating the Hurst exponent

To estimate the Hurst exponent (*H*) of a long-memory process, a power law must be identified in the second order statistics (such as the variance) of the dataset. The relationship between β and *H* depends on the method used to derive the power law (13–15).

Recent research has shown that a reliable and robust estimate of *H* is possible with the use of wavelets (15–17), designed to isolate activity at different frequencies and timescales (14, 15). Recursively applying a wavelet transform with dyadic sampling to a dataset yields wavelet coefficients *d_m_*(*n*) at each time scale *m*. The larger the *m*, the lower the frequency that *d_m_*(*n*) describe, with the highest frequency occurring at *m* = 0, that is, at the sampling frequency. *H* is estimated by identifying the power-law exponent β that occurs in the *scalogram*, that is, the plot of *m* versus the variance of wavelet coefficients,

$$y_{m}=\frac{1}{N_{m}}\sum_{n=1}^{N_{m}}|d_{m}\left(n\right){|}^{2}-{\hat{g}}_{m}.$$

The parameter ${\hat{g}}_{m}\approx -\frac{1}{N_{m}\ln2}$ is a correction factor used to so that the estimate of *H* is unbiased. When this method is used, the Hurst exponent *H* is computed as *H* = 0.5(β + 1). The variance at each *m* is given by $\sigma_{m}^{2}\approx -\frac{2}{N_{m}\ln^{2}2}$ and can be used to infer the confidence of the estimate. For stationary processes, the estimated values of *H* range between 0.5 and 1. When *H* = 0.5 is found, the underlying process cannot be determined: it may infer a true system with *H* = 0.5, that is, with no memory between events, but may also result from systems with long memory but unpredictable variance. Our methods fail to identify the type of memory involved. When 0.5 < *H* < 1, there is evidence of memory in the system, and thus there is a correlation between past and present events. The higher the *H*, the longer this memory is (15, 16).

**Figure 2:**
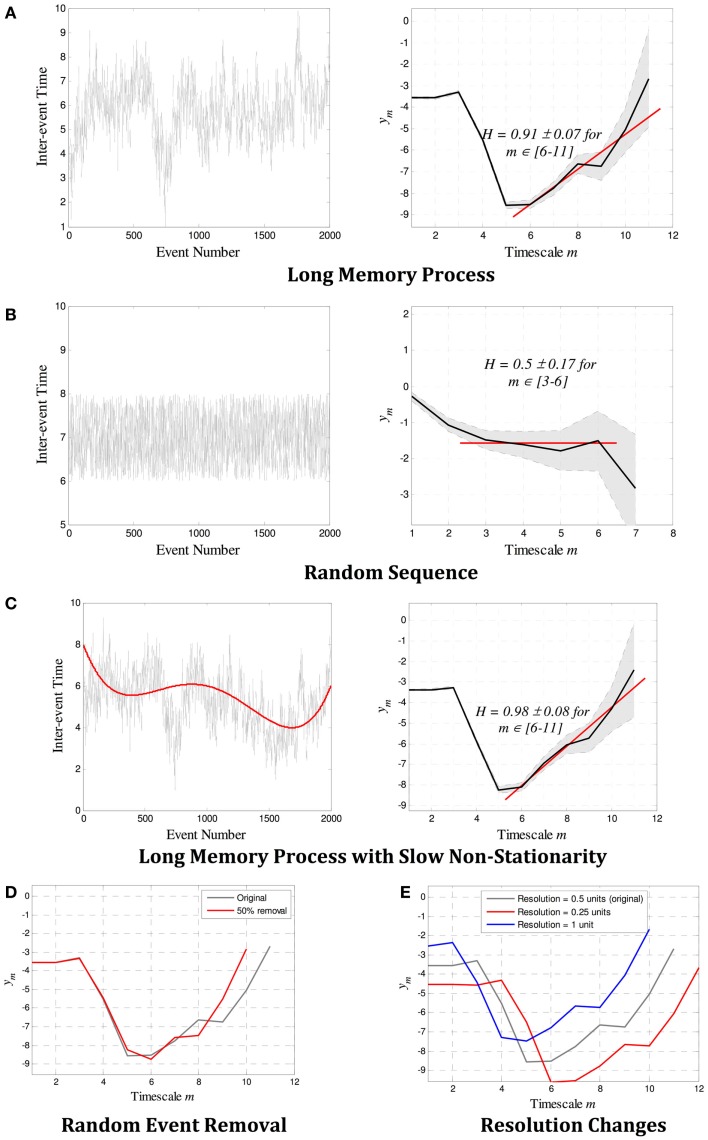
**Example estimates of the Hurst exponent *H* for (A) a long-memory process with *H* = 0.9, and (B) a random process with *H* = 0.5**. In each case, the *scalogram* (a log–log plot of *m* versus *y_m_*) shows a region of alignment of at least four scales that correctly identifies a power law with gradient β and *H* = 0.5(β + 1). **(C–E)** show the robustness properties of wavelet estimation tools. The gradient β (and therefore *H*) is not affected by **(C)** slow non-stationarity, **(D)** a large number of missing events, or **(E)** the resolution of the point process. In **(A–C)**, the 95% confidence limits as defined by the variance $\sigma {}_{m}^{2}$ at each scale is denoted by the gray shaded region between dotted lines, and the red line shows the gradient β identified over the region of alignment. The error bounds and the linear fit are not shown in **(D–E)** for easier visualization, though they are similar in magnitude and quality as those in **(A–C)**.

Example estimates of *H* for both random and long-memory processes are shown in Figures 2A,B. Figures 2C–E show that wavelet estimation tools are robust in the presence of smooth non-stationarity, accurate even when a large number of events are removed, and not affected by the resolution of the records. More detailed description of the methods can be found in the on-line methods section.

## Results

### The existence of power law

**Figure 3:**
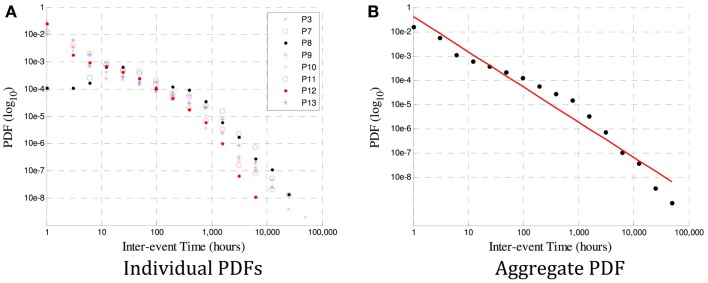
**In (A) are the PDF distributions (in a log-log plot) for each of P1–8**. A power law is evident, but the gradient for each subject varies, and estimates of the scaling exponent β may be influenced by insufficient data in some subjects. The aggregate PDF in **(B)** shows an estimated power law with gradient β = − 1.5. The deviation from linearity that occurs at ~1000 h is also observed at a different time in ~10% of the subjects in Ref. (18), and could be caused by insufficient data at large time scales (leading to an under-estimate of β) or by a genuine excursion from a true power law in the data.

In Figure 3A, it is the probability density function (PDF, or normalized histogram) of the inter-seizure times of each of the eight viable datasets. It is evident that a power law likely exists in all datasets, although the variability in the number of events used to derive individual PDFs would make an estimate of each slope error-prone. So as to obtain a better estimate, the events of all eight subjects were grouped together to derive a combined PDF, shown in Figure 3B.

### The existence of long-memory processes

**Figure 4:**
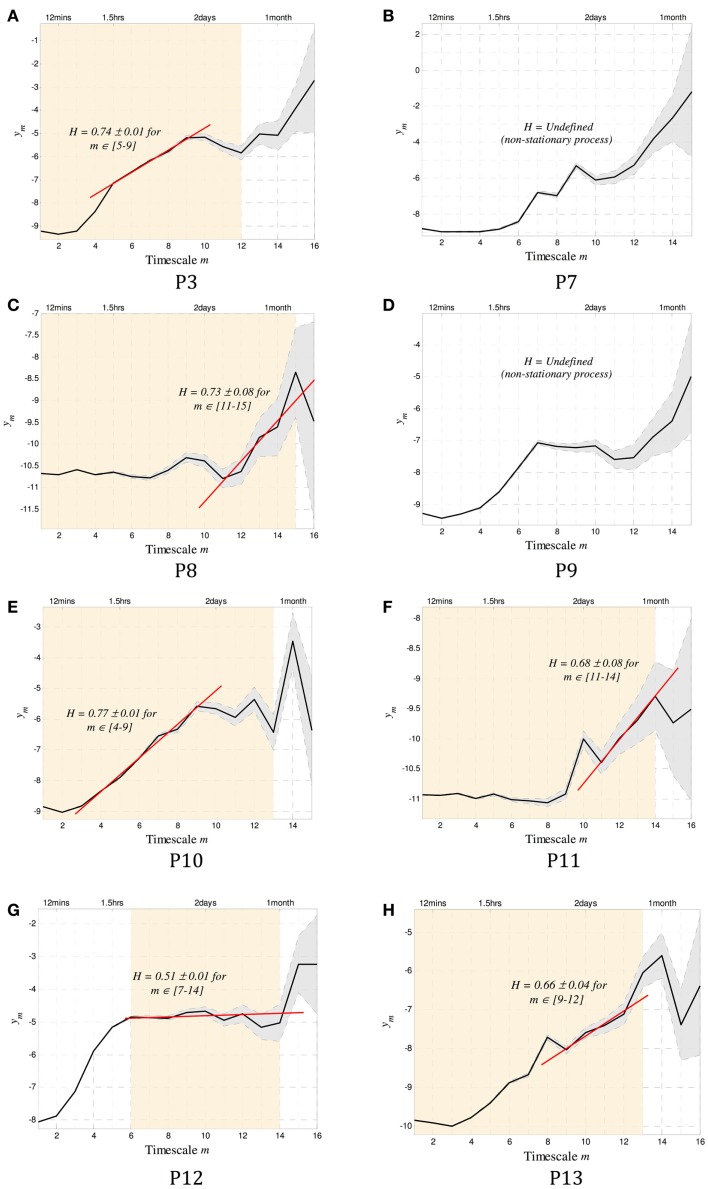
**The scalograms for P1–8 are shown**. In addition to the conventions used in Figure 1, the orange shaded background denotes scales over which the data were stationary and that can be used to estimate *H*. Of the eight subjects, all but two (P2 and P4) showed stationary scales from which a Hurst exponent *H* could be computed. Of the remaining six subjects, five were found to have regions of alignment with scaling exponents consistent with the existence of long memory, with *H* ranging from 0.66 to 0.77. The last subject (P7) showed potentially random correlations between time scales (*H* = 0.51 ± 0.01). Note that to infer stationarity for P7, the data were divided into three segments containing ~1500 events each. At the small time scales, two of the segments agreed with the results, and one did not. This may imply a sharp dynamic change occurring sometimes during the 1.8 years of recording. A summary of all results can be found in Table 2.

The existence of long-memory processes was evaluated for the eight subjects, and the resulting scalograms shown in Figure 4.

The Daubechies wavelet was used for all calculations of *H*. The order of this wavelet transform dictates the level of non-stationarity tolerated in the data. To select an appropriate number, the wavelet order was systematically increased until the resulting scalograms were approximately constant. In all cases, wavelet order 4 was sufficient. To test for more abrupt non-stationarity that is not tolerated by this method, data were divided into segments of approximately 250–500 events (depending on the total length of the record), and the scalograms for each segment were recomputed. The range of scales where the gradients coincide across all segments was identified as the stationary scales for each subject. These are shown as the shaded background of Figure 4. Two subjects (P2 and P4) were completely non-stationary and were not included in further analysis.

To compute the Hurst exponent, regions of alignment were defined as any four or more scales within the stationary regions over which a straight line could be drawn (15). A Hurst exponent consistent with the existence of long memory was found in five out of the six subjects with sufficiently stationary data, with *H* ranging between 0.66 and 0.77. Conservative estimates of the time scales where dependence was evident ranged between 30 min and 40 days (Table 2).

**Table 2:** **Summary of results**.

	P1	P2	P3	P4	P5	P6	P7	P8
Stationary	YES	NO	YES	NO	YES	YES	YES	YES
Long Memory found	YES	–	YES	–	YES	YES	NO	YES
Hurst exponent *H*	0.74 ± 0.01	–	0.73 ± 0.08	–	0.77 ± 0.01	0.68 ± 0.08	0.51 ± 0.01	0.66 ± 0.04
Region of Alignment (scales *m*)	5–9	–	11–15	–	4–9	11–14	7–14	9–12
Length of Dependence (time)	1 h–1.5 days	–	3–40 days	–	30 min–1.5 days	3–20 days	4 h–20 days	17 h–6 days

## Discussion

We have confirmed previous findings of a power-law relationship in inter-seizure intervals, on a large and unique human dataset. A scaling exponent of β = − 1.5 was shown to exist in the averaged PDF of all eight subjects (Figure 3B), consistent with earlier finding (18). Wavelet-based tools were used to compute a *patient-specific* estimate of the Hurst exponent *H*. In five subjects, *H* was consistent with the existence of a long-memory process, while in one subject the correlation between seizures was indeterminable and potentially negligible. This estimate was shown to be robust even when the length of the data is relatively short and when the likelihood of inaccurate records (in the form of missing events) is high. To our knowledge, this is the first time that long-range memory has been identified in human seizure frequency, and similarly the first time a Hurst exponent has been estimated.

The advantage of using wavelet tools to identify the existence of a power law (and, in this case, the existence of long memory) is that the scaling exponent β can be computed on a patient-specific basis. Traditional methods of estimating β require very high quality data over a very long period of time for a robust fit (19). Data must often be aggregated over many subjects, leading to conclusions that are only relevant on average and losing any patient-specific information (e.g., related to differing pathology or site of origin). This is demonstrated in Figure 3, where the variability in exponent between subjects in (a), as well as the deviation from a true power law in some cases (e.g., P3) are lost when the aggregate β = − 1.5 estimated in (b).

A range of time scales that are involved in these long-memory processes can be identified using this method of estimating *H*. For example, in P3 dependence exists from ~3 to 40 days, implying that the dynamics that led to the generation of a seizure are affected by events that took place in the range up to 40 days prior.

In one subject (P7), no dependence could be identified in the stationary region of the scalogram (*H* = 0.5). It is possible that some dependence exists at smaller time scales, but the process was not sufficiently stationary to make for a robust estimate at those scales.

There are a number of potential limitations to this study. The patient group suffering refractory seizures may not be typical, and the medication used in treatment, as well as routine changes in doses made during the study, may have effects on the timing and occurrence of events not considered in our analysis. Data dropouts through brief telemetry failure may also have influenced our findings, but these were not felt to be significant, and modeling indicates that random loss of data, even at high rates, does not affect our estimates (see Figure 2D).

There was a marked discrepancy between seizure frequencies as estimated from patient diaries and those captured by the implanted system. While this was to some degree accounted for by a high number of sub-clinical (type 3) events, both the clinically reported (type 1 events) and clinically confirmed but unreported (type 2 events, as ascertained through analysis of event-triggered audio recordings) were still often orders of magnitude greater than expected, confirming the poor reliability of subjects ability to recognize events.

Existence of long memory is useful in the analysis and interpretation of signals such as the EEG. In the study of epileptic seizure prediction, over 20 years of research has yielded a few success stories (20–22), but none have been readily generalized to wider datasets. The existence (or otherwise) of long memory could explain the differences in performance between different people – if long memory is present then the underlying dynamics are relatively less complex than one where memory cannot be established. In effect, the system becomes more predictable. As an example, we can look at the prediction algorithm proposed in Cook et al. (11), which uses the same data as in this study. Of the 8 subjects included here, the seizures of P2, P4 and P7 were unpredictable. Interestingly, we have shown that the dynamics of P2 and P4 are non-stationary, and that only random (or alternatively, highly complex) correlations exist between events in P7. A long-memory process was identified for all other subjects, and between 54 and 100% of seizures were correctly predicted for these subjects. Although the dataset is limited, it appears that the existence of memory between events is necessary for successful prediction.

The results presented here can be applied in algorithm design directly – it is now known, in a patient-specific way, the dependence of epileptic seizures on past events, and data that reflects this should be included (or accounted for) in any predictive analysis. When analyzing the dynamics of epilepsy, we have shown that an appropriate mathematical model should incorporate mechanisms that allow for interactions stretching far into the past, and that the length of dependence should be tunable.

These findings also have implications for the clinical management of epilepsy. Many authors have remarked on the inadequacy of current methods of assessing the efficacy of anticonvulsants, exclusively based currently on patient diaries (7, 23). More sophisticated methods of analysis based on better understanding of the non-random occurrence of events may permit recognition of treatment effects at a much earlier time point (4). Determination of the optimum time for medication withdrawal after a suitable seizure-free interval may also be more accurately estimated (24). The findings here do not, however, confirm Gower’s dictum that “seizures beget seizures” (25), with progressive escalation in the frequency of events, but rather that there is a deep structure to the timing and occurrence of seizures, with a complex inter-relationship between past and future events.

## Conflict of Interest Statement

Mr. David Himes and Mr. Kent Leyde were employees of Neurovista Corporation at the time the data was acquired. The other co-authors declare that the research was conducted in the absence of any commercial or financial relationships that could be construed as a potential conflict of interest.
